# Tuberculosis of the Cuboid: A Case Report

**DOI:** 10.7759/cureus.72974

**Published:** 2024-11-04

**Authors:** Samriddhi Sarkar, Vishnu Harikrishnan, Shivam Sahu

**Affiliations:** 1 Department of Orthopedics, Mahatma Gandhi Medical College and Research Institute, Sri Balaji Vidyapeeth, Pondicherry, IND

**Keywords:** anti-tubercular therapy, cuboid bone, granulomatous inflammation, mycobacterium tuberculosis, osteoarticular tuberculosis, tuberculosis

## Abstract

Tuberculosis (TB) is a major global health concern, with skeletal TB representing a small fraction of total cases. Foot involvement is relatively uncommon in skeletal TB, and infections of the cuboid bone are especially rare. This case report describes an 18-year-old male who presented with pain and swelling in the dorsolateral left midfoot. A CT-guided biopsy and histopathological examination confirmed necrotizing granulomatous inflammation due to Mycobacterium tuberculosis. The patient was treated with a six-month course of anti-tubercular therapy (ATT) and strict non-weight-bearing protocols, leading to significant symptom improvement. This report emphasizes the importance of histopathological confirmation in diagnosing rare forms of TB and supports existing treatment protocols.

## Introduction

Tuberculosis (TB) remains a significant global health challenge, ranking as the second leading cause of death from infectious diseases worldwide, following COVID-19 (World Health Organization, 2023). Thirty high-TB burden countries accounted for 87% of the world’s TB cases in 2022, and two-thirds of the global total was in eight countries: India (27%), Indonesia (10%), China (7.1%), the Philippines (7.0%), Pakistan (5.7%), Nigeria (4.5%), Bangladesh (3.6%), and the Democratic Republic of the Congo (3.0%) [1]. While pulmonary TB is the most common form, extrapulmonary manifestations, including skeletal TB, are also notable. Skeletal TB is relatively rare, accounting for about 1-2% of all TB cases, with foot involvement being even less frequent, representing approximately 10% of skeletal TB cases [2]. Among these, tuberculosis of the cuboid bone is particularly uncommon. This case report highlights an instance of cuboid tuberculosis, focusing on diagnostic challenges, treatment outcomes, and a comparison with existing literature.

This article was previously presented as a poster at the 2023 OASISCON in Mangalore on September 2, 2023.

## Case presentation

An 18-year-old male presented in January 2023 with a three-month history of pain and swelling localized to the dorsolateral aspect of the left midfoot. The pain was insidious in onset, progressively worsening, exacerbated by walking and alleviated by rest and analgesics. The associated swelling was progressively increasing, firm, non-compressible, and fixed, measuring 4x4x2 cm. The patient reported no constitutional symptoms or comorbidities.

**Figure 1:**
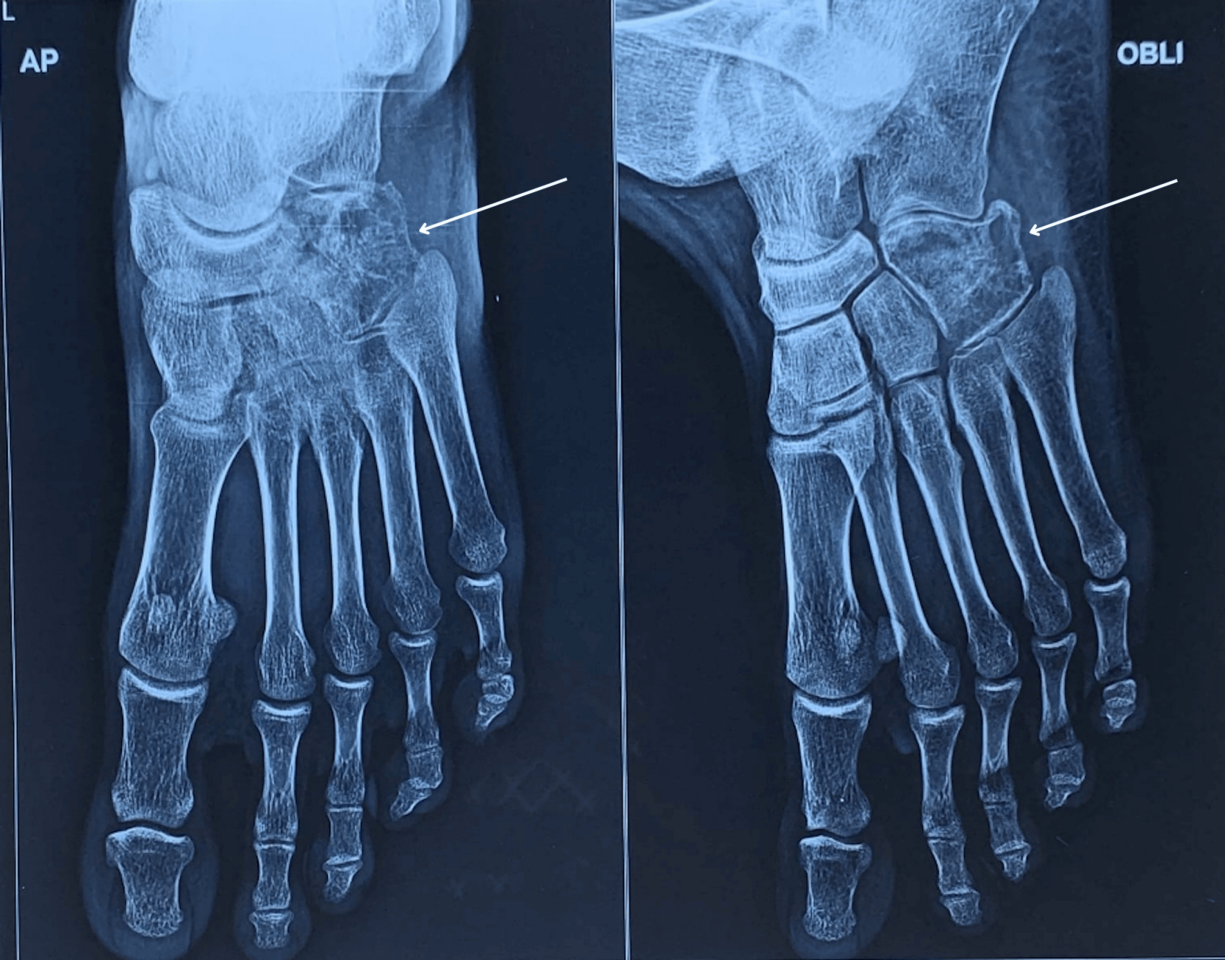
Radiograph of left foot (anteroposterior and oblique views) on presentation showing osteolytic lesion of cuboid.

**Figure 2:**
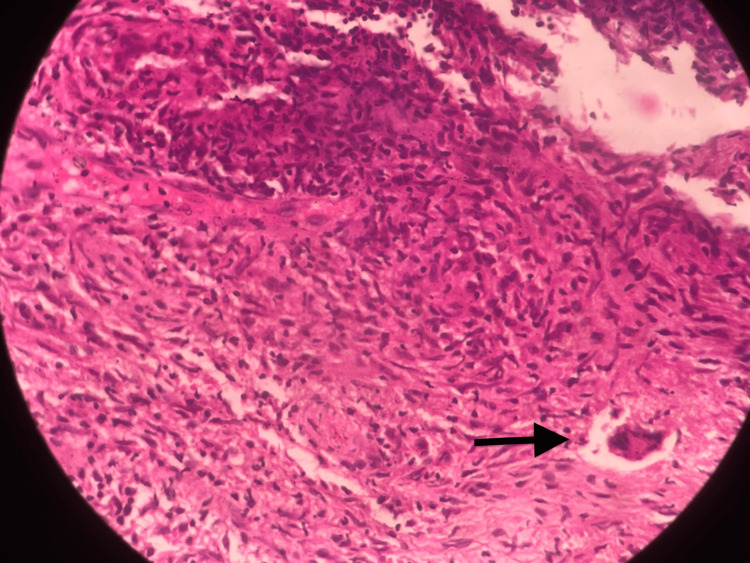
Histopathological image of specimen from CT-guided biopsy of the lesion from left cuboid showing necrotizing granulomatous inflammation, suggestive of tuberculosis.

**Figure 3:**
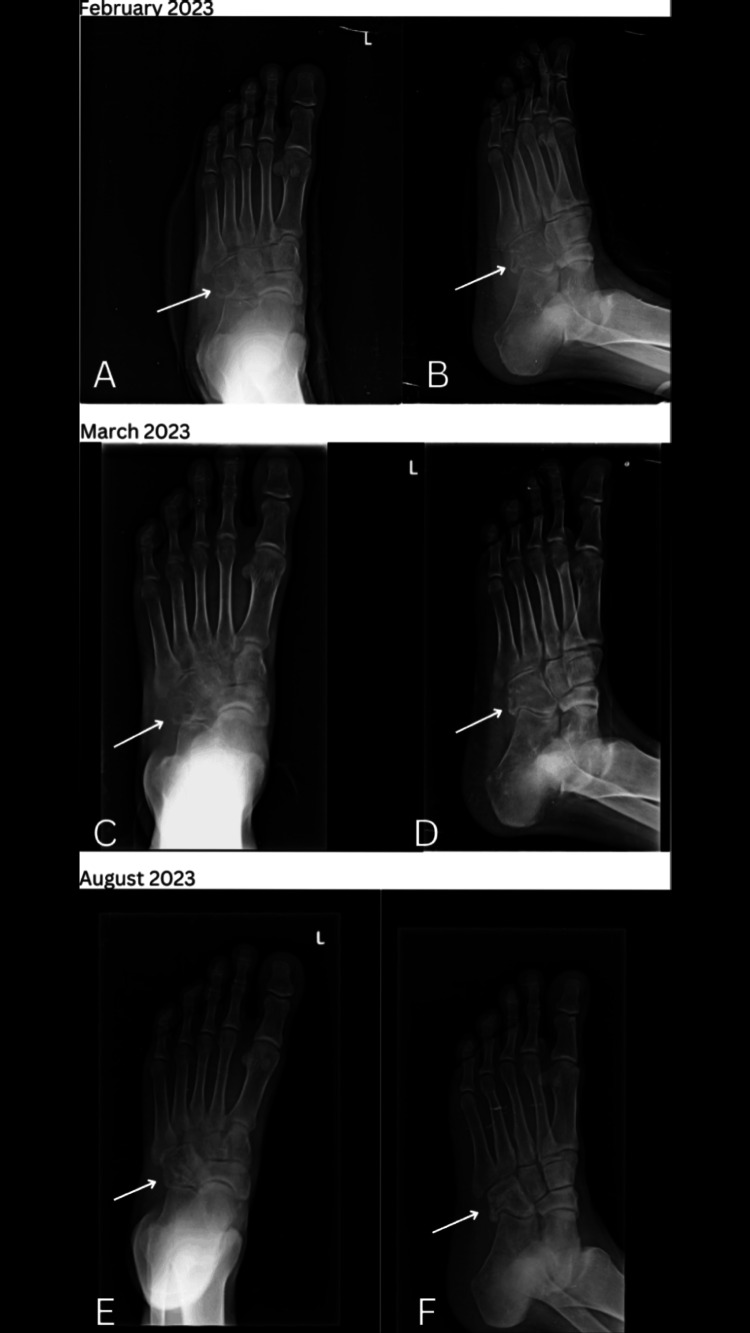
Serial radiographs following the start of anti-tubercular therapy (ATT) showing resolution. A, B: radiograph of cuboid lesion after one month of ATT; C, D: ongoing resolution of cuboid lesion after two months of ATT; E, F: improvement in cuboid lesion after completion of ATT ATT: anti-tubercular therapy

Physical examination revealed a firm, non-compressible, and fixed swelling over the lateral midfoot, with tenderness but no warmth. Initial radiological evaluation suggested a differential diagnosis of chondroma or giant cell tumor (GCT) of the cuboid (Figure 1). A CT-guided biopsy indicated granulomatous inflammation, raising suspicion of tuberculosis. Histopathological examination confirmed necrotizing granulomatous inflammation (Figure 2). An open biopsy and cartridge-based nucleic acid amplification test (CBNAAT) subsequently confirmed the presence of rifampicin-sensitive Mycobacterium tuberculosis. The patient was started on anti-tubercular therapy (ATT) and adhered to a strict non-weight-bearing regimen for one month, followed by partial weight-bearing and full mobilization. After completing a six-month course of ATT, the patient experienced a significant reduction in pain and swelling and is currently asymptomatic (Figure 3).

## Discussion

Osteoarticular tuberculosis is typically secondary to a primary pulmonary or extrapulmonary TB focus, often with an obscure primary lesion. Foot involvement, though rare, presents particular challenges due to the interconnected nature of midfoot joints, including the Lisfranc and subtalar joints [3]. Our case of cuboid tuberculosis aligns with the literature indicating that while foot TB is uncommon, its occurrence necessitates careful diagnostic consideration due to its potential for widespread disease in interconnected joints. The diagnosis of tuberculosis of the foot can be challenging as TB mimics other pathologies radiologically. The initial differential diagnosis included tumors such as chondroma or GCT, which underscores the importance of histopathological confirmation in such cases. The biopsy revealed necrotizing granulomatous inflammation, consistent with TB, confirming findings reported by Procopie et al., who highlighted the critical role of tissue biopsy in distinguishing TB from other conditions [4].

Our patient’s response to a six-month course of ATT is consistent with the treatment protocols outlined in the literature. The standard treatment for skeletal TB involves a multi-drug regimen, including isoniazid, rifampicin, ethambutol, and pyrazinamide [5]. The effectiveness of ATT in alleviating symptoms and achieving clinical remission is well documented, as evidenced by the resolution of symptoms and radiological improvement in our patient. This aligns with findings from Davidson and Horowitz et al., who reported successful outcomes with ATT in skeletal TB cases, including rare instances involving the foot [6].

The rarity of cuboid TB is reflected in the literature. As per a literature search on skeletal TB, only 14.1% involved the foot and ankle, with even fewer cases specifically affecting the cuboid. This highlights the unusual nature of our case and the need for heightened clinical awareness. Additionally, studies emphasize that while skeletal TB is less common in developed regions, its incidence remains higher in countries with prevalent TB rates and socio-economic factors conducive to TB reactivation [7].

Approved methods for diagnosing TB include culture, direct microscopy, biomolecular tests, and whole genome sequencing; however, their widespread use is often limited due to costs, available resources, time constraints, and operator efficiency. Efforts are underway to optimize these diagnostic methods and to develop new techniques. Selecting an appropriate drug regimen relies on the susceptibility profile of the detected isolate. Currently, 16 new TB drugs are undergoing evaluation in phase I or II clinical trials, with an additional 22 in preclinical stages. Many of these new drugs are oral, and shorter treatment regimens are also being tested. The goal of these shorter regimens is to improve patient adherence and prevent relapse or further drug resistance. Screening for TB infection, particularly among vulnerable populations, offers an opportunity for intervention before the disease progresses to an infectious stage. New regimens are also being assessed for the effectiveness of shorter treatment durations in this population. Additionally, there is significant research into post-exposure vaccinations for this group. Global collaboration and the exchange of expertise are critical in the ongoing effort to eradicate TB worldwide [8].

Our case contributes to the understanding of cuboid tuberculosis by demonstrating successful management through a standard ATT regimen and strict adherence to non-weight-bearing protocols. This case reinforces the importance of considering TB in the differential diagnosis of midfoot lesions and supports existing literature on effective treatment strategies for skeletal TB manifestations.

## Conclusions

Cuboid tuberculosis, though an exceptionally rare form of skeletal TB, presents significant diagnostic and therapeutic challenges due to its infrequency and similarity to other musculoskeletal conditions such as tumors. This case of an 18-year-old male with cuboid TB highlights the critical role of thorough clinical examination, radiological imaging, and, most importantly, histopathological confirmation through biopsy in establishing an accurate diagnosis. The use of CBNAAT and histopathology helped differentiate TB from bone tumors like chondroma or giant cell tumors, guiding appropriate treatment. Early initiation of multi-drug anti-tubercular therapy (ATT), coupled with strict adherence to a non-weight-bearing regimen, resulted in significant clinical improvement. This case reinforces the importance of considering tuberculosis in the differential diagnosis of persistent midfoot lesions, particularly in TB-endemic regions. Moreover, it emphasizes that skeletal TB, while rare, can be successfully treated with standard ATT protocols, resulting in favorable patient outcomes. The report adds valuable insights into the diagnosis and management of rare skeletal TB manifestations and serves as a reminder of the global burden of TB, especially in developing countries where such cases are more prevalent. Continued awareness and vigilance are essential in diagnosing and managing unusual presentations of TB to prevent long-term complications and ensure timely, effective treatment. 
